# *Humulus lupulus* L. Strobilus In Situ Photosynthesis and Respiration Temperature Responses

**DOI:** 10.3390/plants12102030

**Published:** 2023-05-19

**Authors:** William L. Bauerle, Michael Hazlett

**Affiliations:** Department of Horticulture and Landscape Architecture, Colorado State University, Fort Collins, CO 80523, USA; mike.hazlett@rams.colostate.edu

**Keywords:** bracts, carbon autonomy, flowering crops, hop cone, Q_10_, organ respiration

## Abstract

The primary metabolism and respiration of the hop strobilus has not been quantified in response to daily temperature fluctuations. The objective of this study was to assess strobilus gas exchange, specifically the response to temperature fluctuations. Hop strobilus were measured under controlled environment conditions to assess the organ’s contribution to carbon assimilation and respiration during the maturation phase. Strobilus-specific daily carbon budgets were estimated in response to temperature fluctuation. The optimal temperature for net carbon gain occurred at 15.7 °C. Estimated strobilus carbon uptake decreased approximately 41% per 5 °C increase in temperature above 20 °C. Daily temperatures within 10–27 °C resulted in a net positive strobilus daily carbon balance, whereas temperature increases beyond 27 °C increasingly exhaust strobilus carbon reserves. The Q_10_ temperature coefficient (the rate respiration increases every 10 °C rise in temperature) approximately doubled per 10 °C rise in temperature from 7–40 °C (1.94–2) with slightly reduced values at lower temperatures. In conclusion, we show that photosynthetically active bracts maintain a positive strobilus carbon balance at moderate temperatures and as mean daily temperatures progressively exceed 27 °C, strobilus net carbon reserves are precipitously exhausted due to ever-increasing respiration rates.

## 1. Introduction

In *Humulus lupulus* L. (hops), the seedless female inflorescence, commonly called a cone, consists of green bracts, bracteoles, and a strig (rachis). Hop cones, which are catkins similar in shape to coniferous cones, arrange their leaf-like bracts and bracteoles around the central rachis. Collectively, these hop cone components contribute to whole cone photosynthesis and respiration; separately, the bracts, bracteoles, and rachis account for approximately 92, 0.3, and 7.7 percent of whole cone biomass, respectively. Relative to a hop leaf, the contribution of the green strobilus components to whole cone photosynthesis and respiration should be considered.

Hop cone quality and yield are known to be sensitive to temperature fluctuation [1,2]. In the two reports describing photosynthesis and respiration in hop cones [3,4], the authors find the photosynthetic contribution of cones to their own carbon demands is significant, however, environmental conditions are a central facet of cone respiration and photosynthesis. Photosynthesis and respiration are temperature sensitive. Photosynthesis presents a thermal optimum between about 10 and 34 °C, whereas respiration rates generally double every 10 °C within this temperature range [5]. The respiratory processes primarily control the net photosynthesis (A_n_) thermal optimum and become increasingly responsible for reduced A_n_ as temperature elevates [6,7,8].

Annually produced plant organs, such as hop cones, have been found to account for 50–80% of daily aboveground respiration [9]. However, by the end of the growing season hop cones account for half of the total aboveground dry matter [9,10]. Studies investigating in situ hop cone photosynthesis and respiration responses to temperature have not been conducted. Understanding hop strobilus responses to temperature would provide insight into their photosynthesis and respiration reaction to temperature fluctuations and the short- to long-term influence on cone CO_2_ exchange.

Our primary objective was to examine the strobilus photosynthesis and respiration response to temperature. Elevated temperatures restrict hop production to select microclimates. Understanding hop strobili physiological responses to temperature is necessary for predicting how increases in temperature will impact strobilus carbon fluxes. We hypothesized that temperature plays a key role in hop cone carbon sequestration. To test the hypothesis, we examined cone temperature responses from 7 to 40 °C. Cone photosynthesis and respiration temperature dependence observations were used to estimate the mean daily temperature effect on cone carbon balance.

## 2. Results

### 2.1. Instantaneous and Daily Strobilus Net Photosynthesis and Respiration Temperature Responses

**Figure 1:**
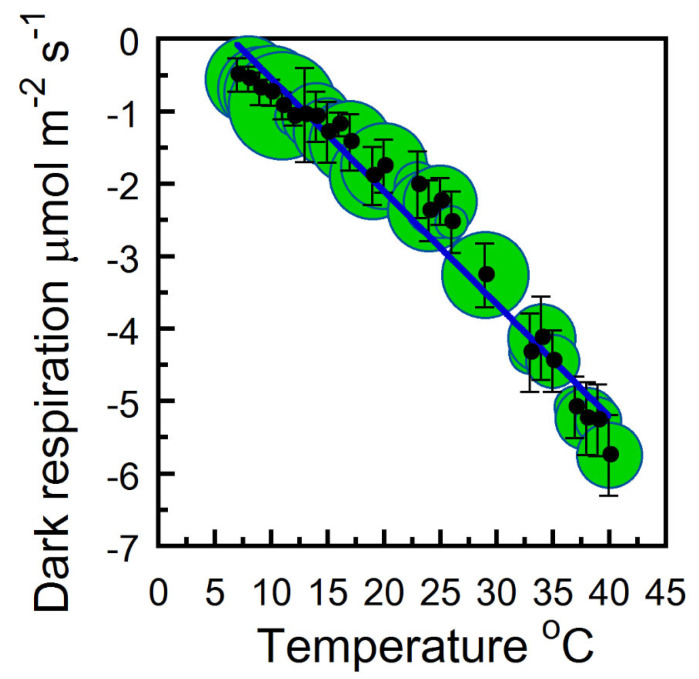
The dark respiration of hop strobili as a function of temperature. Cuvette O_2_ and CO_2_ were atmospheric ambient (~21% and 415 μmol mol^−1^). Samples were pooled across four measurement intervals (n = 24; black circles are means ± SE). Strobili temperature measurements were binned per 1 °C as indicated by green bubble diameter (n = 2–33). The Solid blue line is a linear regression fitted to the entire data set (0.274 × x + 1.09; r^2^ = 0.96).

**Figure 2:**
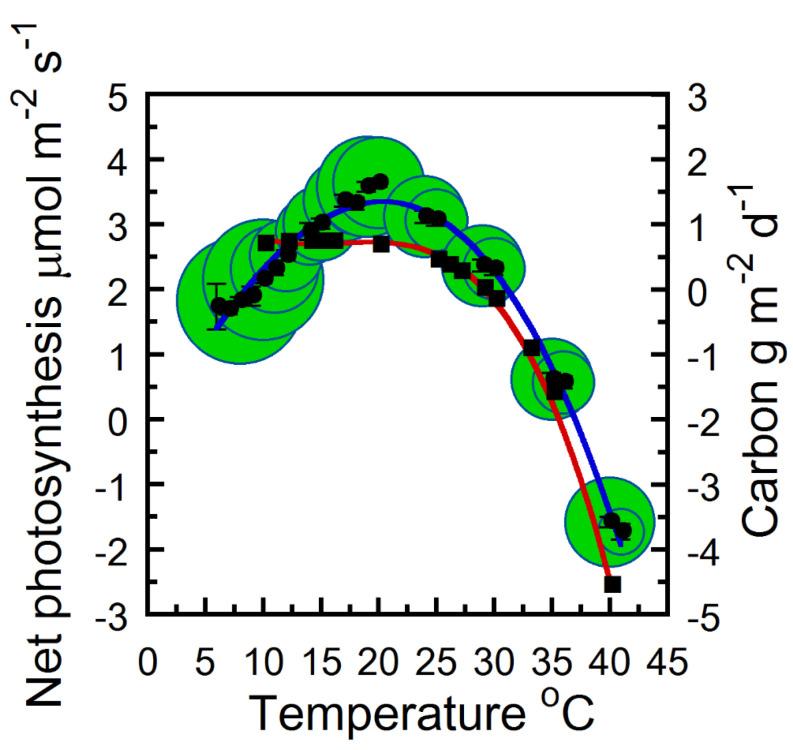
The net photosynthesis and carbon gain and loss responses of hop strobili as a function of temperature. Cuvette O_2_ and CO_2_ were atmospheric ambient (~21% and 415 μmol mol^−1^). Photosynthetically active radiation was controlled at 400 μmol m^−2^ s^−1^. Samples were pooled across four measurement intervals (n = 24 means ± SE). Strobili temperature measurements were binned per 1 °C as indicated by bubble diameter (n = 2–43). Solid circles are the mean instantaneous net photosynthesis (μmol m^−2^ s^−1^) and solid squares are the daily carbon estimates (g m^−2^ d^−1^). Solid blue line is a second order polynomial regression fitted to the entire observed data set (−8.71 × 10^−5^ × x^3^ + −5.32 × 10^−3^ × x^2^ + 0.326 × x + −0.34*;* r^2^ *=* 0.99). Solid red line is a second order polynomial regression fitted to the daily carbon estimates (−4.26 × 10^−4^ × x^3^ + 2.14 × 10^−2^ × x^2^ + −0.349 × x + 2.56*;* r^2^ *=* 0.99).

We observed a decline in leaf dark respiration rate (R_d_) as strobilus temperature decreased from 40 to 7 °C (Figure 1). The mitochondrial R_d_ relationship with temperature was best described by a linear function (Figure 1; r = 0.96). The rate R_d_ respiration increases every 10 °C rise in temperature, also known as the Q_10_ temperature coefficient, remained relatively constant. Q_10_ approximately doubles per 10 °C rise in temperature from 7–40 °C (1.94–2). At 25 °C, strobilus mitochondrial R_d_ was approximately −2.2 ± 0.32 μmol m^−2^ s^−1^. Under strobilus saturated photosynthetically active radiation (PAR) (400 umol m^−2^ s^−1^) and atmospheric ambient CO_2_ (415 umol mol s^−1^), the measured optimum temperature for whole cone A_n_ occurred from 15–20 °C (Figure 2). Observed strobilus carbon autonomy persisted in offsetting R_d_ and photo respiration under light saturated conditions from approximately 7–32 °C at ambient atmospheric CO_2_ (415 umol mol s^−1^) (Figure 2). Analysis of estimated net strobilus carbon gain and loss responses to temperature from 10–40 °C indicated 15.7 °C was the optimal mean daily temperature for maximum strobilus daily net carbon gain, a temperature value in close agreement with the instantaneous measured observations (cf Figure 2 measured vs. estimated values). Likewise, estimated Q_10_ within that temperature 10–40 °C range remained relatively constant and similar to our measured observations (Q_10_ ~ 2). Daily mean temperatures within 10–29 °C resulted in a net positive strobilus carbon balance estimate (Figure 2).

### 2.2. Diurnal Strobilus Carbon Balance Estimates

**Figure 3:**
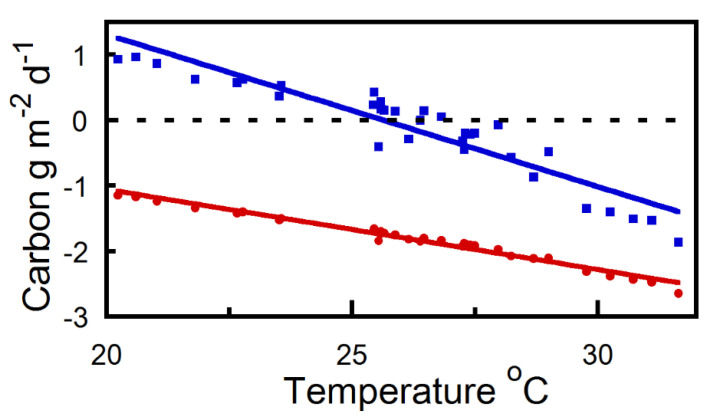
Net hop strobili daily carbon and respiration estimates versus mean daily temperature per m^2^ at Yakima, WA (day of year 205–237, 2022) at ambient (415 μmol mol^−1^) CO_2_. Blue line and squares are daily carbon gain/loss and red line and circles are daily respiration (g m^−2^ d^−1^). The Solid blue and red lines are a linear regression fitted to the entire carbon gain/loss (0.232 × x + 5.96; r^2^ = 0.87) and respiration data set (0.122 × x + 1.39; r^2^ = 0.98).

**Figure 4:**
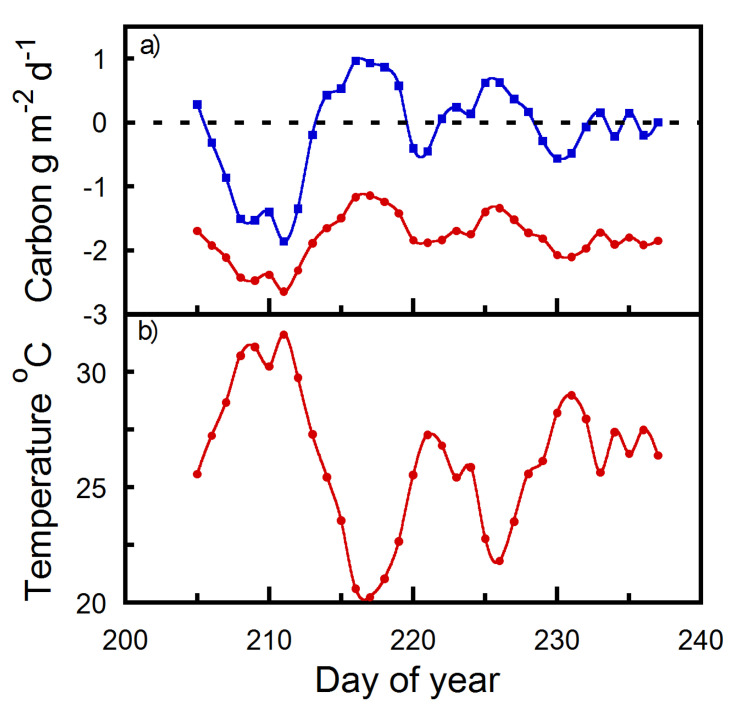
Hop strobili daily carbon gain/loss and respiration estimates per m^2^ at Yakima, WA (day of year 205–237, 2022) at ambient CO_2_ (415 μmol mol^−1^). (**a**) blue line and squares are daily carbon gain/loss and red line and circles are daily respiration (g m^−2^ d^−1^). (**b**) red line and circles are the mean daily temperature (°C).

Apart from our gas exchange measurements not encompassing the earliest strobili growth stage, strobilus maintained a net positive carbon balance under certain environmental conditions. We discovered that strobilus exposed to late season 2022 Yakima, WA weather variables prior to harvest can maintain an autonomous carbon balance when daily mean temperatures do not exceed approximately 27 °C (Figure 3). Figure 4 illustrates summed 30 min diurnal strobilus carbon estimates of net daily carbon per strobilus surface area as described in Section 4.4. Once mean daily temperatures exceed 27 °C under late season 2022 Yakima, WA weather, the increase in strobilus respiration offsets carbon fixation, resulting in a net daily strobilus carbon deficit (Figure 4). As daily mean temperature increased per 5 °C increment above 20 °C, an approximate 41% respiratory offset in net strobilus daily carbon balance occurred (Figure 3). Therefore, late season 2022 Yakima, WA temperature elevations beyond 27 °C increasingly exhausted strobilus carbon reserves (Figure 4).

## 3. Discussion

Numerous studies have provided evidence that the rate at which the nighttime temperature is increasing is greater than the rate at which the daytime temperature is increasing [11,12,13,14]. The increase in nighttime temperature subjects the daily carbon balance of reproductive plant organs, such as hop strobili, to increases in nighttime respiration. Recently, reports confirmed the negative impact of rising nighttime temperature on crop yields (e.g., Chilean quinoa, cotton, rice, and wheat) [15,16,17,18,19,20,21,22]. We observed that elevated temperatures have a pronounced effect on increasing strobilus respiration (e.g., Figure 3). For example, when daily temperatures exceed 27 °C, strobilus daily carbon balance changes from net positive to negative due to increased organ respiration (Figure 4). As compared to hop leaves, strobili have a lower photosynthetic temperature optimum [4,23]. This exacerbates the organ’s sensitivity to elevated temperature relative to leaves [4,23,24]. As a result, carbon loss from the temperature increases can completely negate the daytime carbohydrate fixed by bract photosynthesis (Figure 4), whereas hop leaves achieve maximal carbon assimilation at temperatures of 21 to 39 °C [23]. Future elevated atmospheric CO_2_ concentrations might help partially counterbalance the respiration induced carbon loss via increases in bract photosynthetic rates [4]. Alternatively, the combination of elevated CO_2_ and temperature could result in a net carbon yield decrease as observed in a few other crops e.g., [24].

Respiration is responsible for the carbon loss of up to 70% of daily fixed photosynthate, e.g., [25,26,27]. Increases in temperature elevate respiration and the added energy expenditure leads to lower energy utilization efficiency because energy production is reallocated to maintenance as opposed to growth respiration [28,29]. We found strobilus photosynthesis can counterbalance respiratory carbon loss and sustain a positive daily carbon budget at cool to moderate temperatures (approximately 7–27 °C). Hence, strobilus daily carbon balance estimates at the prevalent hop growing 46–48th parallel north (e.g., Yakima, WA, USA) are generally positive due to the cool to moderate late season daily temperature conditions. However, future elevated nighttime temperatures are expected to have a predominant impact on yield losses [15,28]. Increases in respiratory carbon loss due to temperature increases would further break down strobilus generated assimilates, possibly to a point that strobili no longer function autonomously with respect to their photosynthetic self-generated carbon [4]. Less overall strobilus carbon would in turn decrease lupulin biosynthesis [4].

As atmospheric temperatures rise, our daily process-based model estimates of strobilus photosynthesis and respiration indicate that elevated daily temperatures decrease strobili carbon. One would then expect lupin biosynthesis to decrease due to the recent discovery that lupin production depends on bract photosynthesis [4]. Our estimates are supported by independent methods in other major crop species that consistently show negative temperature impacts on yield [30,31]. Without effective adaptation and genetic improvement to mitigate temperature’s negative effect on hop strobilus photosynthetic activity and respiration, in the future warmer climates are likely to increase strobilus carbon loss and adversely impact hop yield and quality.

## 4. Materials and Methods

### 4.1. Plant Material

Research experiments were conducted at the Colorado State University (CSU) Horticulture Center in Fort Collins, Colorado. Over the course of the study, female tissue culture propagated plantlets were used (Summit Plant Labs Inc., Fort Collins, CO, USA). The public variety ‘Centennial’ was selected due to the representative genetic hop parentage (Brewers Gold + Fuggle + East Kent Golding + Bavarian) [32]. Centennial is ‘ripe to flower’ when ≥20 nodes are visible [33,34].

Plantlets were transplanted into 11 L bato buckets containing 100% horticulture grade perlite. Plant spacing, irrigation, fertilization, and cultural growing condition procedures are outlined in Bauerle [34]. One bine per container was trained to a vertical net trellis at approximately 0.5 m bine length. Initially, all pots were watered to saturation and permitted to drain for 18 h. Thereafter, container moisture capacity was maintained daily. White plastic sheeting was cut and placed on the substrate surface to mitigate evaporation.

### 4.2. Environmental Conditions

Top lights and interlighting bars (suspended horizontally at 150 and 210 cm) (GreenPower LED^®^, Philips Lighting, Amsterdam, The Netherlands) provided supplemental PAR per plant row (100–700 μmol m^−2^ s^−1^ during photoperiod). Daylength was controlled at 18 and 15 h during the vegetative and generative phase. Controllers were programmed to permit the maximum amount of light penetration (shade cloth was only pulled when intense solar radiation and temperatures demanded additional cooling efforts) where daytime ambient PAR at the canopy surface was generally 800–1100 µmol m^−2^ s^−1^. Greenhouse conditions were programmed to a set point air temperature of 26 °C during photoperiod and 20 °C during the dark with a 45 min temperature step change between the two. Daytime temperatures over the experimental period averaged 26.4 °C, but in some instances, temperatures climbed higher despite continuous cooling. Supplemental humidity was provided via an evaporative cooling pad and the daytime saturation vapor pressure deficit (VPD) averaged 1.9 kPa. Air temperature and relative humidity (RH; %) were measured using EHT RH/temperature sensors mounted at the top of the canopy (Meter Devices, Pullman, WA, USA) and PAR using two-line quantum sensors placed parallel to the North/South row orientation adjacent to the plant stems (model LI191R, Li-Cor Inc., Lincoln, NE, USA). A third quantum line sensor was placed 0.5 m above the canopy. The line sensors sampled PAR every minute and then recorded a 15 min average (CR10x; Campbell Scientific, Logan, UT, USA).

### 4.3. Strobili Gas Exchange Measurements

To examine the photosynthetic capacity characteristics, six replicate plants, located in the center of the plot, were sampled for strobili gas exchange traits. Sixteen days after anthesis, ten-day interval repeated measurements began on fully expanded strobili using a portable gas exchange system (CIRAS-2, PP Systems, Haverhill, MA, USA). The whole strobili cuvette was fitted with a full spectrum light-emitting diode and was environmentally controlled (Model PLC C, PP Systems, Haverhill, MA, USA). A PP Systems environmentally controlled conifer branchlet cuvette (Model PLC5 (C)) was used to measure whole strobili as in Bauerle [4]. PAR was controlled with a full spectrum light-emitting diode and temperature by means of Peltier elements fitted with heat sinks and fans (Model PLC5 (C), PP Systems, Haverhill, MA, USA). The measurements were performed at a controlled VPD of 1.5 kPa. A preliminary experiment indicated A_n_ stability after eight minutes per temperature change.

Daytime light saturated PAR (400 μmol m^−2^ s^−1^) and dark (0 μmol m^−2^ s^−1^) strobili temperature response curves were constructed separately at controlled-environment cuvette temperature set points of 7, 10, 12, 15, 17, 20, 25, 30, 35, and 40 °C with eight minutes of stabilization per temperature change. For photosynthesis and respiration calculations, surface bract area (BA) per hop cone was measured and calculated as the total surface area of a cone:

BA = πr (r + l)(1)

where radius (r), and slant height (l) account for the area of the cylinder base and the cone (r^2^ = 0.97, n = 5).

### 4.4. Diurnal Strobilus Carbon Balance Estimates

Photosynthetic CO_2_, light, and temperature response parameters were used to parameterize deterministic strobili-level models for energy balance, stomatal conductance, and photosynthesis as in Bauerle [4]. MAESTRA, a spatially explicit biophysical and biochemical model, estimated the impact of elevated temperature on strobili photosynthesis and respiration. A detailed description of MAESTRA’s equations for carbon assimilation and loss are described elsewhere [22]. A simulation of carbon gain was performed at light saturation and ambient atmospheric CO_2_ to place the cone temperature responses in the context of cone diurnal carbon balance, photoperiod was set at 15 h, the approximate daylength for cone physiological functions in Yakima, WA (46.6021° N, 120.5059° W) at the beginning of August (day of year 213). Half-hourly averages of incident PAR, temperature, RH, and wind speed at Yakima, WA were downloaded from the Washington State University AgWeatherNet site (https://weather.wsu.edu/) (accessed on 17 September 2022). Daily mean atmospheric temperature of 10, 15, 20, 25, 30, 35, and 40 °C were input to estimate strobilus carbon gain responses to typical field daily mean temperatures. Daily simulations of carbon gain were performed at daytime light saturation (15 h) and nighttime dark conditions (9 h). Atmospheric CO_2_ was set to ambient (415 μmol mol^−1^). Q_10_ was calculated at 10 °C increments from 10–40 °C.

Predicted net daily cone carbon gain (C_g_) is a direct estimate of carbohydrate biosynthesis:

C_g_ = (A_n_;light − R_d_;dark) × 12 (2)

where A_n_;light is the total A_n_ during the light period, R_d_;dark is the total respiration during the dark period, and 12 is the molecular mass of C. We summed 30 min diurnal carbon estimates to arrive at net daily C_g_ per strobilus surface area to estimate the impacts of atmospheric temperature on C_g_.

### 4.5. Statistical Analysis

The sample size for A_n_/temperature curves was six replicates (n = 6), repeatedly measured per ten-day interval over the course of four repeated measures (40 days). Each plant was an experimental unit treated as a replicate (n = 6). Two tailed *t*-tests were used to analyze the significance between each combination of repeated measurements. Plant response data were analyzed using SPSS 27 (IBM Analytics, Armonk, NY, USA, www.ibm.com/analytics/ (accessed on 7 November 2022)). Differences between means were considered significant when the *p* value of the *t*-value was <0.05.

## Data Availability

The data presented in this study are available on request from the corresponding author.
